# The role of composite technique in managing peri implant re-fractures in a case with supracondylar fracture of the femur: a case report

**DOI:** 10.4076/1757-1626-2-8174

**Published:** 2009-06-16

**Authors:** Altaf Ahmad Kawoosa, Shabir Ahmad Dhar, Mohammed Farooq Butt, Ghulam Nabi Dar, Mohammed Ramzan Mir

**Affiliations:** Government Hospital for Bone and Joint Surgery, Bagat BarzullahSrinagar, KashmirIndia

## Abstract

**Introduction:**

Osteoporosis of the long bones challenges the orthopaedician in several ways. Amongst the difficulties encountered are the reduced bone mass, increased bone brittleness and medullary expansion, which must be factored in when deciding the type of surgical method to be used.

One of the commoner complications of fixation of fractures in such bone is the occurrence of peri implant fractures with subsequent management requiring significant surgical acumen and judgment.

**Case presentation:**

We report a case who sustained a supracondylar fracture of the femur, which was managed initially by a dynamic condylar screw assembly. The patient sustained a peri implant fracture during her rehabilitation, which was managed by the application of a longer side plate. 4 months postoperatively the patient sustained another peri implant fracture. Using a minimally invasive method we removed the screws from the plate and passed an intramedullary implant. The whole assembly was rotationally stabilized using an Ilizarov fixator. The union proceeded uneventfully.

**Conclusion:**

Use of such minimally invasive methods can be beneficial in such complicated situations.

## Introduction

Osteoporosis of the long bones challenges the orthopaedician in several ways. Amongst the difficulties encountered are the reduced bone mass, increased bone brittleness and medullary expansion, which must be factored in when deciding the type of surgical method to be used [1].

The compromised bone strength and synonymous bone fragility are the consequence of either excessive bone resorption resulting in decreased bone mass and micro-architectural deterioration of the skeleton or an inadequate formation response to increased resorption during bone remodeling [2].

Peri implant fractures of the femur are mostly reported around the prosthetic implants [3].

When a plate is applied to bone, the modulus of the plated section is higher than the rest of the bone and there is an abrupt transition from the plated to the unplated bone. This stress riser often is the site of fracture of the plated bone.

We report a case where the supracondylar fracture in the porotic bone was plated twice and sustained refractures at the interface. To avoid the problems of further devitalisation, extensive exposure and consequent complications we used a minimally invasive method combining intramedullary fixation and the ilizarov fixator as a neutralizing and locking device.

## Case presentation

A 60-year-old Kashmiri female presented to our department after having sustained a fracture of femur in the supracondylar region. After initial management, the fracture was fixed with a dynamic condylar screw assembly. After an uneventful postoperative period, the patient was discharged with advice to undergo supervised physiotherapy. 3 months into the postoperative period, the patient reported to our emergency department with pain in the thigh. X rays revealed a fracture above the dynamic condylar screw at the plate bone interface. The patient was admitted and revision surgery with a longer barrel plate performed. The patient followed up for a period of 4 months and was bearing weight when she again had pain in the peri implant area. Radiographs revealed a fracture of the femur at the new peri implant region. The patient's femoral neck radiograph revealed an osteoporosis grading of 3 according to Singh's classification [4]. Keeping in view the potential morbidity associated with the repeat plating we planned a method that would be less invasive and less destructive to the local fracture environment. The proposed surgery was explained to the patient and her attendants. The ethical board permission was sought and obtained. The patient was taken to the operating room and placed on a fracture table. The screws in the plate were removed percutaneously under image intensifier control to ensure the patency of intramedullary canal leaving the plate insitu to avoid reexposure of the bone surface. The fracture was reduced and fixed with a reamed antegrade intramedullary nail. Anticipating the difficulties in distal locking with an insitu plate an external locking device comprising of a distal full ring and a proximal Italian arch connected by two threaded rods were used. No blood transfusion, bone grafting were used and the mean operating time was 40 minutes. The patient was ambulated on the first post operative day and discharged from the hospital on the same day after advising range of motion exercises of the knee. The patient was advised to compress the fracture at a rate of 1 mm per day in divided increments. The follow up was done at two week intervals. At a mean follow up of 8 weeks when the signs of healing were seen on the radiographs the external ilizarov fixator was removed. The fracture united at 14 weeks with a mean range of motion of the ipsilateral Knee being 0 to 120 degrees. There were no complications associated with the procedure.

## Discussion

The fact that osteoporosis causes and aggravates fracture treatment is well known. Osteoporosis is defined as a skeletal disorder characterized by compromised bone strength predisposing an increased risk of fracture, according to the NIH consensus statement 2000 [5].

Plates act as stress shielding devices where the implant causes the creation of a stress riser at the end of the plate. This was the case with our patient whose initial fracture united in both the plating procedures but a new fracture was created at the stress riser. This problem is especially difficult in the osteoporotic bone. The elderly population is particularly vulnerable to low energy peri implant fractures attributed to osteopenia or osteoporosis leaving limited reconstruction options to the revision surgeon. In our case, it was difficult to envisage a modification in the initial surgery. Retrospective analysis indicated that use of a GSH nail at the outset might have prevented these complications [peri implant fractures].

In planning treatment in older patients with peri implant fractures of the osteoporotic bone, several important factors are to be considered. The functional demands of the elderly are different from young healthy and long term immobilization in bed must be avoided. Delaying treatment has been reported to increase mortality [5].

Our patient was particularly difficult to manage in view of the failure of the DCS method on two occasions. We did not go for a revision with the same implant due to one previous failure. Removing the implant and doing intramedullary fixation would mean denudation and devitalisation besides causing large amount of blood loss. The fact that a prior fracture is associated with an 86% increased risk of new fracture indicates that osteoporosis persists during the treatment of the first fracture [7].

The advantages of our method include,

1. No need of re-exposure and bone grafting
2. The benefits of closed reduction and a reamed nail
3. Easy locking by the external ilizarov device
4. Compression across the fracture by the Ilizarov device.

The disadvantages of an external fixation if any are negated by its limited period of application; we strongly recommend this procedure for such complicated fractures. However in employing such a procedure it is important for the surgeon to be well versed with intramedullary nailing as well as the Ilizarov methodology.

**Figure 1.**
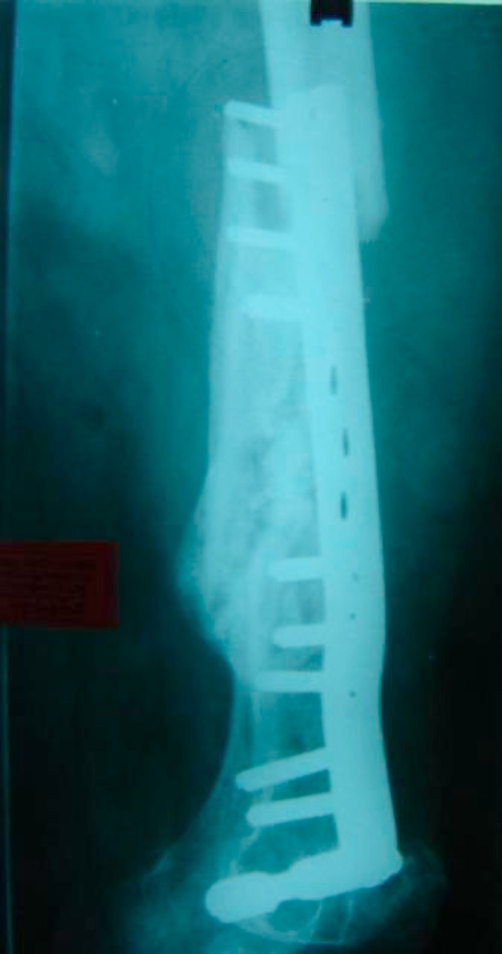
X ray showing peri implant fracture. The previous peri implant fracture was bridged by this plate.

**Figure 2.**
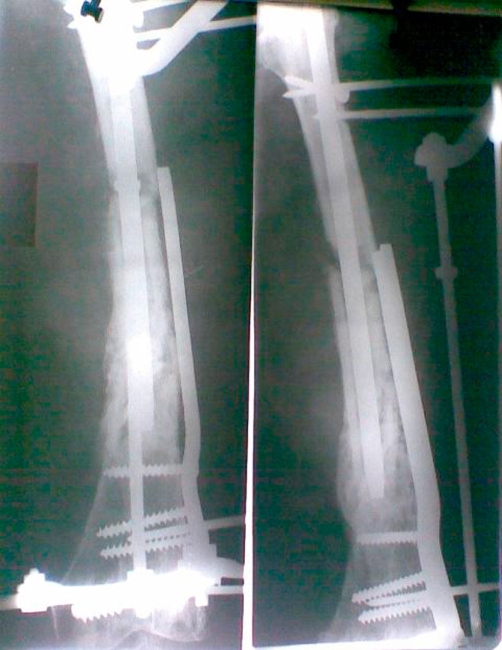
X ray after fixation.

**Figure 3.**
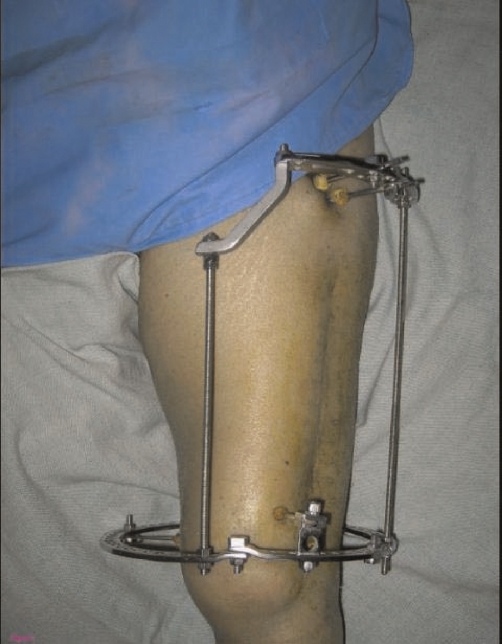
Cinical photograph with Ilizarov external fixator in place.

**Figure 4.**
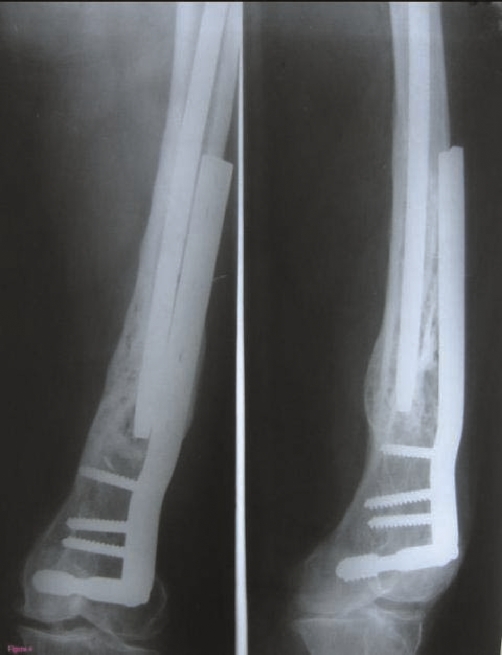
Final X ray showing union of the fracture.

## Conclusion

Use of such minimally invasive methods can be beneficial in such complicated situations.

## Abbreviations

None.
